# Evaluation of Parasiticide Treatment with Benznidazol in the Electrocardiographic, Clinical, and Serological Evolution of Chagas Disease

**DOI:** 10.1371/journal.pntd.0004508

**Published:** 2016-03-14

**Authors:** Abilio Augusto Fragata-Filho, Francisco Faustino França, Claudia da Silva Fragata, Angela Maria Lourenço, Cristiane Castro Faccini, Cristiane Aparecida de Jesus Costa

**Affiliations:** Dante Pazzanese Institute of Cardiology, Vila Mariana, São Paulo, Brazil; Instituto de Ciências Biológicas, Universidade Federal de Minas Gerais, BRAZIL

## Abstract

**Introduction:**

Chagas disease is one of the most important endemic parasitic diseases in Latin America. In its chronic phase, progression to cardiomyopathy has high morbidity and mortality. The persistence of a normal electrocardiogram (ECG) provides a similar prognosis to that of a non-diseased population. Benznidazole (BNZ) is the only drug with trypanocidal action available in Brazil.

**Materials/Methods/Results:**

A group of 310 patients with chronic Chagas disease who had normal ECGs at the first medical visit performed before 2002 were included. There were 263 patients treated with BNZ and 47 untreated. The follow-up period was 19.59 years. Univariate analyses showed that those treated were younger and predominantly male. As many as 79.08% of those treated and 46.81% of those untreated continued with normal electrocardiograms (p <0.0001). The occurrence of electrocardiographic abnormalities and relevant clinical events (heart failure, stroke, total mortality, and cardiovascular death) was less prevalent in treated patients (p <0.001, p: 0.022, p: 0.047 respectively). In multivariate analyses, the parasiticide treatment was an independent variable for persistence of a normal ECG pattern, which was an independent variable in the prevention of significant clinical events. The immunofluorescence titers decreased with the parasitological treatment. However, the small number of tests in untreated patients did not allow the correlation of the decrease of these titers with electrocardiographic alterations.

**Conclusion:**

These data suggest that treatment with benznidazole prevents the occurrence of electrocardiographic alterations. On the other hand, patients who develop ECG abnormalities present with more significant clinical events.

## Author Summary

Twenty years of follow-up of patients with Chagas disease treated with benznidazole is presented in this paper. The persistence of a normal electrocardiogram (ECG) provides a similar prognosis to that of a non-diseased population. Benznidazole (BNZ) is the only drug with trypanocidal action available in Brazil. A group of 310 patients with chronic Chagas disease who had normal ECGs at the first medical visit performed before 2002 were included. There were 263 patients treated with BNZ and 47 untreated. The occurrence of electrocardiographic abnormalities and relevant clinical events (heart failure, stroke, total mortality, and cardiovascular death) was less prevalent in treated patients. In multivariate analyses, the parasiticide treatment was an independent variable for persistence of a normal ECG pattern, which was an independent variable in the prevention of significant clinical events. The immunofluorescence titers decreased with the parasitological treatment. However, the small number of tests in untreated patients did not allow the correlation of the decrease of these titers with electrocardiographic alterations. These data suggest that treatment with benznidazole prevents the occurrence of electrocardiographic alterations. On the other hand, patients who develop ECG abnormalities present with more significant clinical events.

## Introduction

Chagas’ disease (CD), described by Carlos Chagas in 1909[1], and caused by a parasite–*Trypanosoma cruzi*, is one of the most important endemic diseases in Latin America, where there are 10 million people infected (about two million in Brazil). The vectorial transmission has historically been the most important. The disease may also be conveyed by blood transfusion, be congenital, or be transmitted orally (this is the most prevalent today in Brazil), among other types of transmission[2][3]. With globalization, many Latin Americans migrated to other continents, carrying this disease and transmitting it through blood transfusion to the inhabitants of non-endemic countries. Therefore, CD is now present in North America, Europe, Asia, and Oceania, and is becoming a worldwide public health problem[4].

After contamination, the acute phase occurs, characterized by severe inflammation and intense parasitemia, although with limited clinical impact and low mortality. This phase lasts for approximately 8 to 10 weeks, followed by the chronic phase with a decrease of parasitemia and inflammation, but not to extinction. Sixty to 70% of patients remain in the indeterminate form (positive serum reaction, no clinical signs, normal electrocardiogram, normal Chest X-ray, normal esophagogram, and normal barium enema). A total of 40 or 30% evolve to clinical forms, with isolated or concomitant heart, esophagus, and colon involvement[2].

The electrocardiogram (ECG) is a very important tool in monitoring patients with CD. Maguire et al[5], following a population of CD patients for seven years, showed that those who maintained a normal ECG, evolved in a similar way to individuals without the disease. This simple test has important prognostic value, and usually is sufficient for clinical follow-up[6][7].

The parasite's role in the chronic phase remains unclear, even one hundred years after the description by Carlos Chagas[8][9][10]

Parasiticide treatment is controversial as to its indication in the chronic phase and as to its real benefits. The criteria for assessing the possible medication benefits and certainty of a cure are not unanimous among authors. Benznidazole is the only drug in Brazil with proven parasiticide action. It is available in 100 mg tablets and the dose recommended for acute patients or children, is 10 mg/kg/day for 60 days of treatment, and in the chronic phase, 5 mg/kg/day, also for 60 days. Major side effects are dermatitis, which occurs in 30% of cases, and polyneuropathy, which is less prevalent. Patients usually tolerate well the side effects described. Significant leukopenia and liver damage are rarely observed, and the occurrence of agranulocytosis is exceptional[11][12][13][14].

The BENEFIT study that randomly evaluated the treatment with BNZ in 2854 (1431 BNZ and 1423 placebo) patients with chronic Chagas cardiomyopathy, NYHA class I, II, III, (97% class I and II), followed for the short period of 5.4 years, showed a significant decrease in parasitemia (PCR test) in the BNZ group. However, no difference in the occurrence of events during this period (death, resuscitated cardiac arrest, insertion of a pacemaker, or an implantable cardioverter–defibrillator (ICD), sustained ventricular tachycardia, cardiac transplantation, new heart failure, stroke or transient ischemic attack, or a systemic or pulmonary thromboembolic event)[15].

Immunofluorescence titers are stable in untreated patients. However, treated patients showed a decrease of the titers. Negativity of the immunofluorescence titers is infrequent, but may occur persistently after more than a decade of treatment[16][17].

This is a retrospective study that analyzes the electrocardiographic, clinical, and serological evolution of patients with chronic Chagas’ disease, with or without treatment with BNZ, and who had a previous normal ECG.

## Materials and Methods

In our database, we evaluated patients with CD confirmed by two or more serum reaction techniques (immunofluorescence, hemagglutination, direct agglutination, and enzyme-linked immunosorbent assay—ELISA). All subjects needed to have a normal ECG at the first medical visit at the institution, done before 2002. The decision to include patients who had had their first visit before 2002 was due to the fact of having a minimum follow-up of 10 years. There was no age limit for inclusion because all patients who satisfied the inclusion criteria were evaluated.

Most of them did not undergo a digestive tract examination (barium swallow or barium enema). Therefore, we did not refer to them as having the indeterminate form, but as asymptomatic patients without heart disease. In the loss of follow-up cases, we attempted to find them by phone, telegram, through social networks, and contact with neighbors or family, which is part of the institution's usual approach to call patients for medical visits when necessary. We do not have the success percentages in each category because there are no specific records.

The ECG of the last visit needed to have been between 2011 and 2013 to have a minimum electrocardiographic evolution of 10 years. In 2013, data collection was ended.

When there was no electrocardiographic tracing during this period, the patient was invited to do so, and those who agreed signed the Informed Consent Form (ICF). The report of the first and last electrocardiographic tracings was done without patient identification or date of execution by the Dante Pazzanese Institute of Cardiology Section of Electrocardiography, according to the Guidelines for Electrocardiographic Analysis and Reports Issued by the Brazilian Society of Cardiology – 2009 [18].

While examining the medical records, patients who presented with significant co-morbidities that could influence electrocardiographic alterations during evolution were not included, such as:

- High blood pressure (HBP) with left ventricular hypertrophy (LVH)
- Coronary or peripheral atherosclerotic disease, with clinical claudication, angina and/or equivalent
- Valve disease with significant clinical manifestation

Some patients remained untreated and others received BNZ at a dose of 5 mg/kg/day for 60 days. This treatment took place before 2002. The attending physician, without prior standardization, made treatment indications. If the patient had received BNZ and discontinued treatment due to a side effect, he would remain in the treated group, as per the principle of intention-to-treat.

The first and last titers of quantitative immunofluorescence of the patients were compared using trypomastigote forms of the parasite [19] (values above 1/40 are considered positive). The quantitative immunofluorescence method is used systematically in patients treated with BNZ. Only this reaction was available.This serum reaction is part of the clinical follow-up routine of these patients. A new collection of a blood sample was requested when there was only one immunofluorescence test, and in patients who agreed signed the ICF.

In all patients included in this study, we analyzed:

- Age (mean and standard deviation) in years
- Sex (male and female)
- Ethnicity (white and non-white—according to the medical record notes)
- Heart rate (HR) in beats per minute
- Systolic (SBP) and diastolic (DBP) blood pressure in mmHg
- Diabetes mellitus (DM), dyslipidemia (DLP), or coronary artery disease (CAD) described in clinical records
- Treatment with BNZ (yes or no)
- Follow-up period at the institution, in years (from the first to the last visit in untreated patients and from the beginning of the treatment until the last visit for those treated with BNZ)
- The exact time when patients definitively left the endemic area—in years
- Comparison between the first (not necessarily pre-treatment) and last titers of quantitative immunofluorescence in patients treated with BNZ.
- Occurrence of relevant clinical events (described in the medical records): heart failure (HF) with clinical or supplementary tests (chest X-ray, echocardiogram), stroke, death from any cause or death from heart disease.

For the statistical analysis, the SPSS software version 19 was used. The quantitative variables were described by mean and standard deviation. To compare quantitative variables, the Mann-Whitney test was used for variables with non-Gaussian distribution. Qualitative variables were presented as absolute and relative frequency (%). The analysis of the relationship between the variables used Fisher's exact test or chi-squared. The "t" test was not used because the variables did not follow a normal distribution in the Kolmogorov-Smirnov test.

Univariate logistic models were made for each variable and those with a significance level less than 0.15 were included in the multivariate model.

Logistic regression was done considering:

1. As dependent variable, the occurrence of combined outcomes (HF, stroke, total mortality) and as independent variables, treatment with BNZ, persistence of normal ECG, follow-up time, males, Caucasian ethnicity, and current age.
2. As dependent variable, the persistence of a normal ECG and independent variables, treatment with BNZ, follow-up time, the male sex, Caucasian ethnicity, and current age.

The accepted significance level was 95%.

### Ethics statement

This study is registered at the Dante Pazzanese Institutional Ethics Committee.

All adult patients gave written informed consent to participate in the study.

## Results

From a database with approximately 1500 patients with CD, 527 had a normal ECG at their first medical visit. Of these, 379 met the inclusion criteria. Three hundred and ten patients were found (81.80%), and 69 (18.20%) could not be reached and were therefore excluded. Table 1 shows baseline characteristics of the group of patients found and the group not found.

**Table 1:** Baseline characteristics of patients found and not found.

	FOUND	NOT FOUND	p
	310 pac (81.80%)	69 pac (18.20%)	
HR bpm	75.14 (± 11.08)	75.04 (± 9.19)	0.719
SBP mmHg	124.79 (± 18.55)	127.39 (± 17.79)	0.629
DBP mmHg	79.82 (± 9.53)	79.93 (± 9.25)	0.958
DM	24 (7.70%)	1 (1.40%)	0.061
DLP	85 (27.40%)	2 (2.90%)	< 0.0001
CAD	7 (2.30%)	3 (4.34%)	0.398
AGE years	38.43 (± 10.30)	38.06 (±8.48)	0.701
MALE	107 (34.50%)	27 (39.10%)	0.488
WHITE	231 (74.50%)	50 (72.50%)	0.761
OEA	17.16 (± 7.71)	16.63 (± 5.50)	0.632
BZ	263 (84.80%)	58 (84.10%)	0.854

HR: heart rate beats per minute; SBP: systolic blood pressure; DBP: diastolic blood pressure; DM: diabetes mellitus occurrence; DLP: dyslipidemia occurrence; CAD: coronary artery disease; OEA: out of endemic area; BZ: treated with benzonidazole

The presence of DLP (described in the medical records) was more prevalent in the group of found patients than in the group not found, with 27.40% and 2.90%, respectively (p <0.001). It was not possible to detect a statistically significant difference between groups for other variables.

We followed the 310 patients included in the study for a period of 10 to 46 years (19.59 ± 6.46), with a median of 18 years; 50% of these patients were followed for 15 to 23 years. Age at the last visit varied from 30 to 84 years (57.80 ± 10.07). Only 107 (34.52%) were male, and 231 (74.52%) were white. Two hundred and sixty-three patients (84.84%) received BNZ and 47 (15.16%) did not. The characteristics of the two groups are shown on Table 2.

**Table 2:** Characteristics of the untreated and treated with BZ groups.

	UNTREATED	TREATED	p
	(47p – 15.16%)	(263p – 84.84%)	
AGE (last visit) years	68.89 (± 6.81)	56.07 (± 9.59)	< 0.0001
FOLLOW- UP years	19.68 (± 8.51)	17.97 (± 5.99)	0.486
MALE	10 (21.30%)	97 (36.9%)	0.045
WHITE	37 (78.70%)	194 (73.80%)	0.586
OEA	19.65 (± 8.69)	16.77 (± 7.49)	0.012
DM	3 (6.40%)	21 (8.00%)	>0.999
DLP	8 (17.00%)	77 (29.30%)	0.109
CAD	2 (4.30%)	5 (1.90%)	0.288
HR bpm	68.32 (± 9.74)	70.32 (± 8.84)	0.198
SBP mmhg	134.32 (± 20.05)	130.07 (± 18.12)	0.202
DBP mmhg	81.36 (± 12.50)	81.30 (± 10.39)	0.716
NL ECG	22 (46.81%)	208 (79.08%)	< 0.0001

OEA: out of endemic area; DM: diabetes mellitus occurrence DLP: dyslipidemia occurrence; CAD: coronary artery disease; HR: heart rate beats per minute; SBP: systolic blood pressure; DBP: diastolic blood pressure; NL ECG: normal ECG

Treated patients were younger (56.07 years x 68.89 years, p <0.0001), predominantly male (36.90% vs. 21.30%, p: 0.045), had left the endemic area more recently (16.77 years vs 19.65 years, p: 0.012), and 208 (79.08%) maintained normal ECGs, compared to 22 (46.81%) of the non-treated individuals (p <0.0001).

Among the treated patients, 55 (20.92%) had ECG changes, as follows: Right Bundle Branch Block in 21 (38%), nonspecific changes in ventricular repolarization in 20 (37%), and Blockage of the anterior superior division of the left branch in 11 (20%). Among untreated patients, 25 (53.19%) had worsening of the ECG: Right Bundle Branch Block in two (8%), nonspecific changes in ventricular repolarization in four (16%), and Blockage of the anterior superior division of the left branch in six (24%). Other changes detected had low prevalence.

There were no statistically significant differences between groups in the other variables.

The side effects observed in treated patients were dermatitis in 92 patients (34.98%), polyneuropathy in 12 (4.56%), and others (dyspepsia, insomnia, leukopenia less than 4000/mm^3^) in eight (3.04%). Twenty-six (9.89%) patients abandoned treatment due to side effects. The analysis of relevant events (heart failure, stroke, and cardiac death or due to any cause) described in the medical records were HF in eight cases (2.58%), stroke in four (1.29%), and 12 deaths (3.87%), in which six were men and six were women. The date of occurrence of these events was not available; only if they had occurred or not. Therefore, a longitudinal analysis was not possible, so only the logistic regression was done. In six of them it was possible to assume that the cause was due to CD (1.93% of all patients studied). In only one case was it not possible to determine the cause of death.

Among the 80 patients who had worsening of the ECGs, eight (10%) died and among the 230 who maintained normal ECGs, four (1.7%) died (p: 0.002). The cause of death related to CD occurred in five (6.25%) patients with ECG alterations and in only one (0.43%) with a normal ECG (p: 0.001). The eight cases of HF occurred in patients with ECG alterations. Among the four cases with stroke, two (2.5% of 80) had ECG alterations, and two (0.9% of 230) did not (p = 0.274). Combined outcomes (HF, stroke, and death) occurred in 24 cases (7.74%), 16 of them (20%) with ECG alterations and eight (3.48%) with normal ECGs (p <0.0001).

Table 3 shows the occurrence of events in patients untreated and treated with BNZ. It shows that patients treated with BNZ had fewer cardiac deaths and fewer total deaths.

**Table 3:** Patients untreated and treated with BZ and events occurrence.

	UNTREATED	TREATED	p
	(47p – 15.16%)	(263p – 84.84%)	
HEART FAILURE	2 (4.26%)	6 (2.29%)	0.348
STROKE	0	4 (1.50%)	>0.999
DEATHS	5 (10.60%)	7 (2.70%)	0.022
HEART DEATHS	3 (6.40%)	3 (1.10%)	0.047
COMBINED OUTCOMES	7 (14.89%)	17 (6.46%)	0.096

There were two or more results of the immunofluorescence test in 171 patients (Table 4), 11 (6.43%) untreated and 160 (93.57%) treated. These results remained stable in untreated patients (232.72 ± 104.02 and 254.54 ± 93.41), whereas in the treated individuals, the titers decreased (144.90 ± 109.80 and 70.25 ± 74.70: p < 0.0001). In the 112 patients who remained with normal ECGs and without any relevant clinical outcomes, the titers of the first and last reactions were 127.50 (± 104.60) and 63.21 (± 65.95), respectively (p <0.001). Titers decreased 39.93% in patients who had ECG alterations and 50.43% in those with normal ECGs (p: 0.863). The difference in years from first to last serology in those who had ECG alterations was 14.18 (± 4.09) years, and in those who maintained a normal ECG it was 14.04 (± 5.01) years (p: 0. 15).

**Table 4:** Immunofluorescence test (two or more): 171 patients.

Patients	1° test title	2° test title	p
Untreated (11) (6.43%)	232.72 ± 104,02	254.54 ± 93,41	NS
Treated (160) (93.57%)	144.90 ± 109.80 (*)	70.25 ± 74.70	< 0.0001
Normal ECG and no events (112) (70%)	127.50 ± 104.60 (*)	63.21 ± 65.95	< 0.001
Total 171 (100%)			

(*) not necessarily pretreatment

The negativity of the immunofluorescence titer (<1/40) occurred in 60 patients treated with BNZ (37.50%), with an average of 14 years follow-up, and in none of the untreated individuals.

In the multivariate analysis (Table 5) with dependent variables, the occurrence of combined events (heart failure, stroke, and total mortality) and independent variables, treatment with BNZ, follow-up time, males, white ethnicity, and age, it was observed that with the withdrawal of the ECG from this model, the parasiticide treatment was the only protection against events.

**Table 5:** Logistic regression model. Dependent variable: the occurrence of clinical combined outcomes (heart failure, stroke and total mortality) and independent variables: treatment (BZ), follow-up, male, Caucasian and age in years.

CI (95%) O.R.				
	O.R.	Lower Limit	Upper limit	p
TREATED BZ	0.330	0.115	0.947	0.039
FOLLOW-UP	1.046	0.986	1.110	0.138
MALE	2.264	0.878	5.834	0.091
CAUCASIAN	3.025	0.679	13.480	0.147
AGE	1.021	0.965	1.081	0.463

Table 6 assesses another logistic regression model, analyzing the dependent variable, persistence of a normal ECG, with the independent variables, treatment with BNZ, follow-up time, male, white ethnicity, and age. In this model treatment with BNZ and white ethnicity favored the persistence of a normal ECG, while the evolution of time (less than average) favored the appearance of ECG alterations.

**Table 6:** Logistic regression model. Dependent variable: normal ECG maintenance and independent variables: treatment with BZ, follow-up, male, Caucasian and age in years.

CI (95%) O.R.				
	O.R.	Lower Limit	Upper limit	p
TREATED BZ	5.7330	2.5396	12.9420	<0.0001
FOLLOW UP	0.9381	0.8990	0.9789	0.0033
MALE	0.9381	0.8990	0.9789	0.0033
CAUCASIAN	0.9381	0.8990	0.9789	0.0033
AGE	1.0190	0.9886	1.0503	0.2243

## Discussion

The etiological treatment of CD remains a controversial subject due to the lack of well-conducted studies to determine the importance of parasiticide treatment. In the database of our institution, we selected patients who had normal ECGs at the first visit, and they fulfilled the inclusion criteria for this study. Despite an exhaustive search using the available resources, it was not possible to contact 69 patients. Analyzing the two groups (found and not found) by two decades of follow-up, there was no significant difference between them except for the prevalence of dyslipidemia in the group included in this study. Therefore, it would be possible to consider that patients included or not included do not evolve differently, and it was assumed that if all patients were included, the results would not have been different.

The analysis of 310 patients shows that the mean follow-up was nearly two decades (in no case less than 10 years). The majority of patients were treated. This difference is due to the fact that the etiological treatment of patients with CD is a routine in our institution. The decision to indicate treatment was made by each medical doctor with the agreement of the patient. Side effects of BNZ were well tolerated despite the fact that dermatitis is very prevalent in about 1/3 of the cases. This was why treatment discontinuation due to intolerance occurred in a small percentage (9.89%). Literature data shows treatment discontinuation due to side effects between 4% and 30%, depending on the prescribed dose [20]. Viotti et al., in an analysis of 283 patients, found 13% of treatment discontinuation [12].

When we compared the groups treated and not treated with BNZ, we noticed that the treated patients were younger. This is due to the previous observation that the older patients who had normal ECGs, rarely presented with electrocardiographic alterations, and therefore, these patients might not have received treatment with BNZ. The prevalence of males among the treated patients was interpreted as a casual observation. Treated patients had definitively left the endemic area where they lived and had been contaminated more recently. It had already been noted that the shorter the period of time away from the region where the infection occurred, the more the treatment should be indicated, because of the unpredictability of clinical evolution in a period of less than 20 years.

There are few studies about the possible benefits of etiological treatment and most of them are non-randomized or not placebo-controlled. Viotti et al., analyzing 566 patients in a chronic phase of CD and without HF (283 treated with BNZ, 5 mg/kg/day for 30 days and 283 untreated) observed electrocardiographic worsening in 4.2% of treated patients and 14.1% of untreated individuals (p: 0.002), during an average period of 9.8 years of follow-up [12]. In 21 years of follow-up, Fabbro et al. evaluated 54 patients treated with BNZ or Nifurtimox and 57 untreated patients, and noted electrocardiographic worsening in 3.7% of treated patients and 15.8% of the untreated individuals (p <0.05) [21]. In a study of 58 patients, 29 treated with BNZ and 29 untreated, after 13 years of follow-up, Machado de Assis et al. found that patients in the indeterminate form of the disease who received BNZ had clinical deterioration in 17.4%, while 56.5% (p <0.05) of the untreated ones in the same condition worsened [22].

In our study, at the end of nearly two decades of follow-up, 79.08% of the patients treated with BNZ remained with normal ECGs, while only 46.81% of patients untreated remained with normal ECGs. These data are consistent with the literature, suggesting the possibility that the BNZ administered in chronic patients with normal ECGs can prevent the onset of electrocardiographic alterations.

Despite the follow-up of 19.59 years when patients with CD and normal ECGs were analyzed, few relevant clinical events were expected due to the slow progression of the disease [6]. De Lana et al. observed 28 chronic CD patients without treatment, 22 in the indeterminate form, and noticed clinical worsening in 2% per year, whereas global clinical worsening was 0.5% per year when initially in the indeterminate form [23]. Among the patients studied, combined outcomes occurred in 24 cases (7.74%), which is consistent with the literature data, considering that all our patients began the follow-up with normal ECGs.

Patients treated with BNZ annually go through a quantitative immunofluorescence test with trypomastigotes [19]. In this group, only 171 patients had at least two immunofluorescence tests, 160 treated with BNZ and 11 untreated. Zauza et al., analyzing 140 chronic patients without parasiticide treatment after 10 years of follow-up, found a statistically significant increase in immunofluorescence titers in patients with progressive clinical deterioration, especially in the age range of 20–59 years [24]. In our study, titers of immunofluorescence of the 11 untreated patients remained stable after 14 years of follow-up. Evaluating 13 patients treated with BNZ and followed during a period of four years, Andrade et al. observed a decline in antibody titers compared to pre-treatment [25]. In a 13-year follow-up of 58 chronic patients, 29 treated with BNZ and 29 untreated, Machado de Assis et al. did not observe negativity of the antibody titers in any case, but noted falling titers in the treated group, especially those treated in the indeterminate form [22]. Viotti et al. followed for 36 months 53 chronic patients treated with BNZ and 89 untreated, and observed a decrease in antibody titers in 64% of those treated and 21% of those untreated (p <0.001). The negativity of serology occurred in 40% of those treated and in 7% of the untreated patients (p <0.001) [17]. In our study, we observed that the 160 patients treated with BNZ along a period of 14 years between the first and last tests, had a decline from the titers of the first antibody test to the last. Since there were only five patients with ECG worsening and combined events, we could not make a better evaluation of the decrease in titers of these patients. In the group of 112 patients treated whose ECGs remained normal and without any clinical events, the decrease of immunofluorescence titers was significant. Despite this variation, it was not possible to identify through the antibody titers the patients who will eventually have ECG alterations or not. Contrary to the observations of Viotti et al [17], 37.50% of our treated patients and none of the untreated patients showed negative immunofluorescence reactions after 14 years of follow-up.

Most important in the follow-up of patients with CD is the possibility of progression to clinical forms, specifically heart disease. In a 1983 publication, Maguire et al. showed the importance of the ECG in CD. Analyzing 431 patients in the chronic phase during a period of seven years, the authors concluded that individuals younger than 60 years who had normal ECGs had mortality rates similar to those of the healthy population [5].

In our study, CD patients who received BNZ remained with normal ECGs in almost 80% of the cases during a mean follow-up of two decades. In the multivariate analysis that considered the occurrence of significant combined clinical events (HF, stroke, and mortality), treatment with BNZ was an independent variable with statistical significance. In the same logistic regression model, the fact that the white patients had been shown as an independent variable favoring the occurrence of events should be analyzed with caution because of the high prevalence of white ethnicity in our study, and this data was not analyzed with more specific criteria. Finally, in another logistic regression model, treatment with BNZ was the independent variable in maintaining a normal ECG. The white ethnicity in this analysis was an independent variable in maintaining a normal ECG, and a follow-up time shorter than the average was an independent variable favoring the occurrence of ECG abnormalities. The study considered white ethnicity as a confounding factor because in one model it favors the onset of clinical events and in another model it favors the maintenance of a normal ECG. Therefore, its value is questionable.

The data obtained in our study may lead us to suppose that CD parasiticide treatment is beneficial, since the patients prevalently maintained normal ECGs, and this fact is important in a better prognosis. Many papers have been published suggesting these same results, highlighting the importance of the parasite in the maintenance of myocardial inflammation, and the elimination or minimization of its presence should be the approach followed [26].

We would like to emphasize that the randomized, placebo-controlled BENEFIT study that showed equal outcomes between patients who received BNZ or placebo, had an average follow-up period of only 5.4 years, and unlike our study, the BENEFIT study enrolled patients with established heart disease. Our observation of patients with normal ECGs at the first visit and with a follow-up of two decades had a different approach and it cannot be compared with the BENEFIT study.

### Study limitations

The adequate and appropriate assessment of the scientific hypothesis in question should be through a randomized controlled clinical trial, which did not happen in this study. However, the data obtained provides useful information because of the large number of patients evaluated during a two-decade follow-up.

### Conclusions

From the data obtained, it could be suggested that treatment with BNZ prevents the appearance of ECG abnormalities, and patients with normal ECGs have fewer combined events. Treatment with BNZ decreases immunofluorescence titers. Therefore, only 37.5% of the treated patients showed negativity of the immunofluorescence titers.
